# Effect of Dietary Geranylgeraniol and Green Tea Polyphenols on Glucose Homeostasis, Bone Turnover Biomarkers, and Bone Microstructure in Obese Mice

**DOI:** 10.3390/ijms24020979

**Published:** 2023-01-04

**Authors:** Chwan-Li Shen, Jannette M. Dufour, Jonathan M. Miranda, Gurvinder Kaur, Eunhee Chung, Latha Ramalingam, Naima Moustaid-Moussa, Jay J. Cao

**Affiliations:** 1 Department of Pathology, Texas Tech University Health Sciences Center, Lubbock, TX 79430, USA; 2 Center of Excellence for Integrative Health, Texas Tech University Health Sciences Center, Lubbock, TX 79430, USA; 3 Obesity Research Institute, Texas Tech University, Lubbock, TX 79409, USA; 4 Department of Cell Biology and Biochemistry, Texas Tech University Health Sciences Center, Lubbock, TX 79430, USA; 5 Department of Medical Education, Texas Tech University Health Sciences Center, Lubbock, TX 79430, USA; 6 Department of Kinesiology, University of Texas at San Antonio, San Antonio, TX 78249, USA; 7 Department of Nutritional Sciences, Texas Tech University, Lubbock, TX 79409, USA; 8 Grand Forks Human Nutrition Research Center, USDA-ARS, Grand Forks, ND 58202, USA

**Keywords:** bone health, glucose homeostasis, tea, bioactive component, obesity

## Abstract

Previously, we demonstrated that the administration of either geranylgeraniol (GGOH) or green tea polyphenols (GTP) improved bone health. This study examined the combined effects of GGOH and GTP on glucose homeostasis in addition to bone remodeling in obese mice. We hypothesized that GGOH and GTP would have an additive or synergistic effect on improving glucose homeostasis and bone remodeling possibly in part via suppression of proinflammatory cytokines. Forty-eight male C57BL/6J mice were assigned to a high-fat diet (control), HFD + 400 mg GGOH/kg diet (GG), HFD + 0.5% GTP water (TP), or HFD + GGOH + GTP (GGTP) diet for 14 weeks. Results demonstrated that GTP supplementation improved glucose tolerance in obese mice. Neither GGOH nor GTP affected pancreas insulin or bone formation procollagen type I intact N-terminal, bone volume at the lumbar vertebrae, or bone parameters at the trabecular bone and cortical bone of the femur. There was an interactive effect for serum bone resorption collagen type 1 cross-linked C-telopeptide concentrations, resulting in no-GGOH and no-GTP groups having the highest values. GGOH increased trabecular number and decreased trabecular separation at the lumbar vertebrae. GTP increased trabecular thickness at lumbar vertebrae. The GG group produced the greatest connectivity density and the lowest structure model index. Only GTP, not GGOH, decreased adipokines concentrations (resistin, leptin, monocyte chemoattractant protein-1, and interleukin-6). In an obese male mouse model, individual GGOH and GTP supplementation improved glucose homeostasis, serum CTX, and trabecular microstructure of LV-4. However, the combined GGOH and GTP supplementation compromises such osteoprotective effects on serum CTX and trabecular bone of obese mice.

## 1. Introduction

Obesity, type 2 diabetes (T2DM), and osteoporosis have become major global health concerns over the last few decades. Approximately 462 million individuals were affected by T2DM cooresponding to 6.28% of the world’s population, or a prevalence rate of 6059 cases per 100,000, in 2017. It is projected to increase to 7079 individuals per 100,000 by 2030, reflecting a continued rise worldwide [1]. The prevalence of osteoporosis is approximatley 18.3% worldwide; it is more common in females, with a prevalence in females 4 times that of males [2]. By 2050, globally there will be up to approximately 21.3 million hip fracture each year [3]. 

Emerging evidence has shown a negative correlation between obesity and bone metabolism in animal models and in humans. Obesity is caused by excessive accumulation of fat in the adipose tissue and associated increased secretion of pro-inflammatory cytokines, which in turn increases chronic inflammation and affects osteoclast activity [4,5,6]. In a murine model, Cao et al. demonstrated the detrimental effect of high-fat diet (HFD) induced obesity on bone as determined by means of micro-computed tomography (*μ*CT) [4]. There are several mechanisms through which obesity negatively affects bone health. Obesity is associated with chronic inflammation. Increased circulation and tissue proinflammatory cytokines in obesity may promote osteoclast activity and bone resorption through modifying the receptor activator of NF-κB (RANK)/RANK ligand/osteoprotegerin pathway [5]. Excessive secretion of leptin and/or decreased production of adiponectin by adipocytes in obesity may either directly affect bone formation or indirectly affect bone resorption through up-regulated proinflammatory cytokine production [5]. Additionally, high-fat intake may interfere with intestinal calcium absorption and therefore decrease calcium availability for bone formation [5]. Fehrendt et al. further showed that moderately increased body weight does not protect bone architecture from age-dependent degeneration. By contrast, bone microstructure in obese animals is negatively affected and reduced maintenance of cell–cell and cell-matrix contacts, osteoblast and trabecular structure, and collagen-I and osteoid synthesis, results in a negative effect of obesity on bone microstructure [6]. 

There are similarities between obesity and T2DM in bone health, but T2DM seems to have additional harmful effects. Emerging evidence suggests that glycation of collagen in T2DM may directly alter cross-linked bone matrix formation or indirectly affect bone through changes of cellular activity in osteoblasts and bone progenitor cells [7]. In obese individuals with T2DM, increased bone marrow fat may exacerbate mesenchymal stem cell and osteoblast impairment by the release of pro-inflammatory cytokines and free fatty acids from hypoxic adipose tissue. This causes a vicious cycle of chronic inflammation and inhibited osteoblastic activity that results in individuals being more vulnerable to microdamage accumulation, fragility fractures at most skeletal sites, and impaired fracture healing [8]. Scientific developments in the understanding of the molecular mechanisms/pathways involved in obesity and T2DM have opened opportunities in new anti-inflammatory treatment approaches for management of bone health [4,9,10]. As such, dietary bioactive components with high anti-inflammatory and antioxidant properties may have great potential in management of obesity-associated bone deterioration. 

In recent years, the use of dietary supplements, bioactive components, nutraceuticals, and botanicals (collectively called functional foods) have become an alternative approach to prevent or alleviate the complications of obesity/T2DM on bone health by mitigating inflammation and maintaining an oxidant-antioxidant balance. Among dietary bioactive compounds, geranylgeraniol (GGOH), an isoprenoid found in fruits, vegetables, and grains with health benefits, has been shown to improve glucose homeostasis [11], lipid metabolism, and osteogenesis and bone remodeling in animals [12]. Chung et al. recently reported that GGOH at 800 mg/kg in the diet of obese mice for 14 weeks improves glucose homeostasis and bone microstructure, probably via suppression of pro-inflammation and modification of microbiome composition [13]. Besides GGOH, green tea has been proposed to have anti-diabetic properties [14,15]. Ample evidence has shown that green tea and its bioactive components can counteract the deleterious effects of the imbalance between osteoclastogenesis and osteoblastogenesis in vitro [16] and improve bone properties in laboratory animals [17] and one human clinical trial [18], due to green tea’s potent anti-oxidant and anti-inflammatory properties. However, the potential combined effect of GGOH and green tea polyphenols (GTP, bioactive components in green tea) supplementation on glucose homeostasis and bone microstructure in obese mice has never been examined. 

Several studies including ours have shown the difference between mice on a low-fat diet (LFD) compared to a HFD in glucose homeostasis, insulin resistance, and bone parameters. For example, we previously reported that compared to the LFD group, the HFD group had glucose intolerance and insulin resistance in obese male mice [13]. In terms of bone aspects, related to the LFD group, the HFD group had decreased bone strength, trabecular and cortical bone volume and thickness, as well as decreased trabecular bone volume, thickness and number in female rats [19]. We recently demonstrated that compared to the animals in the HFD group, the animals on a HFD supplemented with a high dose of GGOH (800 mg/kg diet) had significantly improved glucose tolerance and bone microstructure [13]. Since comparisons of LFD vs. HFD and HFD vs. GGOH in glucose homeostasis and bone parameters from experiments using the same batch of animals have been reported previously [13], this study was focused on whether GGOH and GTP would additively or synergistically counteract effects of diet-induced obesity in glucose homeostasis, insulin resistance, and bone parameters in a diet-induced obese model. We hypothesized that (a) administration of GGOH in diet plus GTP supplementation in drinking water will additively or synergistically mitigate HFD-induced bone deterioration in male mice, and (b) such changes are related to an improvement of glucose homeostasis and a reduction in inflammation. 

## 2. Results

### 2.1. Glucose Homeostasis and Pancreatic Morphological Analysis

ipGTT values were measured at five different time points. For ipGTT, at 120 min after glucose administration, both GGOH and GTP supplementation significantly improved glucose tolerance in obese mice, as shown by decreased blood glucose (Table 1). Both GGOH and GTP supplementation decreased ipGTT AUC. There was no significant interaction of GGOH supplementation and GTP administration on ipGTT parameters. ipITT values were also measured at five different time points. Both GGOH and GTP supplementation significantly improved insulin sensitivity at 120 min ipITT and GGOH and GTP tended to decrease ipITT AUC (0.05 < *p* < 0.1). Only GTP supplementation, not GGOH supplementation, increased serum insulin concentration in obese mice. However, the pancreas insulin concentration was not affected by either GGOH or GTP supplementation (Table 1). 

**Figure 1:**
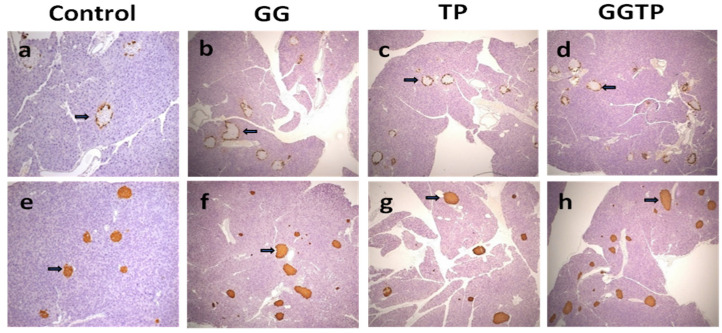
Effect of geranylgeraniol (GGOH) and green tea polyphenols (GTP) on immunohistochemical analysis of pancreatic tissue. Pancreatic tissue sections collected from mice fed HFD (**a**,**e**), High-fat-diet (HFD) only (control group), HFD supplemented with 400 mg GGOH/kg diet (GG group, (**b**,**f**)), HFD supplemented with 0.5% GTP in drinking water (TP group, **c**,**g**), and HFD supplemented with GGOH and GTP (GGTP group, (**d**,**f**)) were immunostained for glucagon (**a**–**d**) or insulin (**e**–**h**) and counterstained with hematoxylin. Group assignment includes control group (high-fat diet group); TP group (high-fat diet and green tea polyphenols at 0.5% (*v*/*w* in drinking water)); GG group (high-fat diet and geranylgeraniol at 400 mg/kg diet); GGTP (high-fat diet, geranylgeraniol, and green tea polyphenols). The arrows in **a**–**d** point to glucagon postivie alpha cells. The arrows in **e**–**h** point to insulin positive beta cells. The magnification is 10×.

Interestingly, regardless of treatment, histological analysis of the pancreas revealed normal exocrine and endocrine tissue with normal arrangement of the glucagon producing alpha cells along the outer edge of the islets (glucagon, Figure 1a–d) and insulin-producing beta cells throughout the islets (insulin, Figure 1e–h). 

### 2.2. Serum Bone Turnover Biomarkers and Bone Microarchitectural Properties

Table 2 lists the effects of GGOH and GTP supplementation on bone parameters. Neither GGOH nor GTP supplementation affected serum P1NP (a bone formation marker) concentration of mice (*p* > 0.05). There was an interaction in serum CTX (a bone resorption marker) concentrations between GGOH and GTP supplementation, resulting in the control group having a greater value of CTX than the other three groups.

With regard to trabecular microstructure, based on the results of two-way ANOVA analysis, GGOH supplementation (the GG and GGTP groups) increased Tb.N and decreased Tb.Sp at LV-4, while GTP supplementation (the TP and GGTP groups) increased Tb.Th at LV-4. There were significant interactions observed in Conn.Dn and SMI at LV-4 (Table 2). Among the four treatment groups, the GG group had the greatest Conn.Dn values and the control group had the lowest values for Conn.Dn at LV-4 (GG group > GGTP group = TP group > control group). Notably, regarding SMI at LV-4, we found that (i) the GG group had the lowest SMI value than the other groups; and (ii) there was no difference in SMI value among control, TP group, and GGTP group, as a result of control group = GGTP group = TP group > GG group. Furthermore, the bone microstructure of the femur mid-diaphysis (cortical bone) was not affected by either GGOH or GTP supplementation (Table 2). 

### 2.3. Final Body Weights, White Adipose Tissue Weights, and Adipokine Concentrations

Table 3 lists the effects of GGOH and GTP supplementation on final body weights, white adipose tissue weights and adipokine concentrations. Both GGOH and GTP supplementation individually resulted in a reduction in final body weights and white adipose tissue weights. Only GTP supplementation, not GGOH supplementation, resulted in lowering the concentrations of adipokines of adipose tissue, including resistin, leptin, MCP-1, and IL-6, in obese mice. There was no interaction of GGOH and GTP supplementation on adipokine concentrations (Table 3). 

## 3. Discussion

In this study, we showed the beneficial effects of individual dietary bioactive components, GGOH and GTP supplementation, on glucose homeostasis and bone microstructure of diet-induced obese male mice. The osteoprotective effect of individual bioactive compounds (GGOH and GTP, respectively) was consistent with our previous findings that (i) GGOH administration at 800 mg/kg diet benefits the bone microstructure and improves glucose homeostasis in obese mice [13] and (ii) GTP supplementation into water at 0.5% (w/vol) improved bone microstructure of rats in obese animals [17]. 

Bone turnover marker changed with GGOH and GTP supplementation. For instance, in the absence of GGOH, the GTP-supplemented group (TP group) had lower serum CTX concentrations than that of non-GTP-supplemented group (control group), while in the presence of GGOH, the lowering effect of GTP was not observed, resulting in no difference between the GG and GGTP groups. Similarly, in the absence of GTP, the GGOH-supplemented group (GG group) had lower CTX concentrations than that of non-GGOH-supplemented group (control group), while in the presence of GTP, the lowering effect of GGOH was not found, leading to no difference between TP and GGTP groups. However, this study did not demonstrate additive effects for mice receiving GGOH in addition to GTP supplementation (the GGTP group). Therefore, the findings of this study on bone remodeling (CTX) do not support our hypotheses that GGOH plus GTP supplementation would have an additive or a synergistic beneficial effect on obese-induced bone deterioration. 

Bone loss is a process caused by the imbalance between osteoblastogenesis and osteoclastogenesis. Excessive reactive oxygen species and pro-inflammatory cytokines induce osteoclastogenesis and suppress osteoblastogenesis [20]. GGOH reduces pro-inflammation and oxidative stress via either inhibiting NF-kB activation [21,22] or activating the mevalonate pathway [23]. The anti-inflammatory actions of GGOH could be contributing to its anti-resorptive effect, as shown by decreased bone resorption biomarker (serum CTX) to the animals in the LFD group in Chung’s study with GGOH at 800 mg/kg diet for 14 weeks in obese mice [13]. However, in the present study, we observed that both GGOH administration and GTP supplementation lowered body weights and white adipose tissue weights, but only GTP supplementation showed the suppression of pro-inflammatory cytokines in obese mice. The non-significant impacts of GGOH on adipokine production were not consistent with our previous report that GGOH supplementation at 800 mg/kg diet decreased adipose MCP-1 and IL-6 concentrations in obese mice. The explanation is probably due to the 50% dosage of GGOH (400 mg/kg diet) used in the present study compared to the published work with GGOH at 800 mg/kg diet [13]. We also speculate that the osteoprotective effects of GGOH on trabecular bone (an increase in Tb.N and a decrease in Tb.Sp) may be exerted via a mevalonate pathway [20], instead of via a suppression of pro-inflammation. Future studies are warranted to address this possible connection. Additionally, the findings of GTP’s anti-inflammatory potential, as shown by a reduction in adipokines in obese mice, agrees with previous work on serum and liver pro-inflammatory cytokines in obese rodents [24,25].

Trabecular bone is a connected bone microstructure. Connectivity, the number of closed loops in the trabecular bone network, is a topological measure that attempts to correlate biomechanical behavior with architecture. Conn.Dn can be calculated by dividing the connectivity estimate by the volume of the scanned trabecular bone sample [26]. Low Conn.Dn of trabecular bone has been reported in ovariectomized rats with lower trabecular bone than those in sham rats. Shen et al. demonstrated that the loss of trabecular bone mass and Conn.Dn in ovariectomized rats is restored by combined treatment of parathyroid hormone and estradiol [27]. Previous studies have shown a positive association between Conn.Dn and Tb.N, as well as a negative association between Conn.Dn and Tb.Sp in ex vivo studies [26,28]. On the other hand, SMI, a widely used method, measures the plate- or rod-like geometry of trabecular structures. SMI is calculated by means of three-dimensional image analysis based on a differential analysis of the triangulated bone surface. SMI describes the degree to which the trabecular network follows common plate-lie or rod-lie structural models. An increase in the SMI indicates a reduction of the more desirable plate-like structure of trabecular network [29]. The findings that the combined GGTP group had the lower Conn.Dn value and higher SMI value in obese mice than those in the GG group does not support our hypothesis that GGOH plus GTP supplementation would have an additive or a synergistic beneficial effect on trabecular bone of obese mice. Such observation of Conn.Dn and SMI in the combined GGTP group was consistent with serum CTX, indicating the combined GGTP group compromises the osteoprotective effects on trabecular bone of obese mice seen in the individual GG or TP group. Although it is commonly acceptable that the majority bone of LV is trabecular bone, the unavailable data of cortical bone of LV-4 is a limitation in this study. 

We previously showed that compared to the mice fed a LFD, the mice fed a HFD diet exhibited impaired glucose tolerance and insulin resistance with no increase in serum insulin concentration in obese mice [13]. Additionally, mice on the HFD with a high dose of GGOH (800 mg/kg diet) led to a significant improvement in glucose homeostasis [13]. Thus, in this study we designed a 2 × 2 factorial model to only evaluate GGOH, GTP, and the combined effect in HFD-fed mice, without a comparison to a group fed a low-fat diet. As expected, we observed that GGOH [13], GTP [30], and EGCG [31] individually improved glucose tolerance and insulin resistance in obese mice. However, similar to the effect on serum CTX, neither an additive effect or a synergistic effect was observed in glucose tolerance and insulin resistance in obese mice. In this study, GTP-supplemented mice (the TP and GGTP groups) had an increase in serum insulin concentration. We speculate the elevated serum insulin was probably due to the addition of sweetener in the drinking water in both groups which may have stimulated the pancreatic beta cells to release more insulin. The effect of non-nutritive sweetener on blood glucose, insulin, or gut hormones is controversial with no effect, increased or decreased glucose concentrations or insulin sensitivity being reported [32]. However, we noted that Splenda was only added to the drinking water of the two GTP-supplemented groups but not to the Control or GG groups, which may be a limitation in the interpretation of results. On the other hand, Qian et al. recently reported sucralose (marked as Splenda) at 0.78 mM dose to male Sprague Dawley rats for 4 weeks significantly improved glucose tolerance in the obese rats via upregulating expression of sweet taste receptors and glucose transporters in the small intestine [33]. Based on Splenda concentration in water, daily water consumption, and animal body weight, the Splenda dose in our TP and GGTP groups was approximately 0.078 g/kg body weight, a dose of 1/20 that given in Qian’s study (1.55 g/kg body weight). Nevertheless, it is worthy to include a group of mice feeding HFD and drinking non-nutritive sweetener (Splenda) to investigate whether sweetener affected blood glucose, insulin, and gut hormone in a future study.

Nevertheless, the TP-supplemented groups (the TP and GGTP groups) were able to lower blood glucose concentrations after a glucose challenge. As noted, neither GGOH nor GTP showed any impact on glucagon and insulin in pancreatic tissues. Previously, we reported that there was no difference in pancreatic morphology between the low-fat diet and HFD mice [13] so it is not surprising that there was no difference between the HFD and GGOH or GTP groups, which all showed normal pancreatic islets. Consistent with normal pancreatic beta cell function, there was a significant improvement in glucose homeostasis in the GGOH and GTP treated mice. The observed beneficial effects on glucose homeostasis with regard to GGOH are associated with the ability of GGOH to lower inflammation and modify the gut microbiome [13]. However, further analysis of the mechanisms for GGOH and GTP are warranted. It would be interesting to examine the effects of GTP on the neuropeptides (i.e., kisspeptin), acting as neurotransmitters, involved in appetite control, as well as the relationship between peptides and GTP on glucose homeostasis [34]. 

Published works have demonstrated EGCG (the most abundant catechin in GTP) increased osteogenic differentiation (osteoblastogenesis) of bone marrow mesenchymal stem cells by increasing mRNA expression of osteogenesis-related genes, alkaline phosphatase activity and, eventually, mineralization [16,35]. On the other hand, Singhatanadgit et al. reported that GGOH prevents zoledronic acid-mediated reduction of viable mesenchymal stem cells [36]. We noted that the main results of the present study are based on morphology and biochemical data to show how GGOH and GTP affect glucose homeostasis and bone microstructure, without the corresponding molecular mechanism(s). Therefore, further studies are warranted to investigate the molecular mechanisms for the combination of GGOH and GTP on osteoblastogenesis using mesenchymal stem cells. This study used an established diet-induced obese animal model and showed impaired glucose tolerance, insulin resistance, and compromised bone metabolism [28,37]. The amount of GGOH (400 mg/kg diet) used in this study was much greater than normal consumption from diets in humans (0.5 mg/d) [38]. Nevertheless, the animal study provides scientific evidence and mechanisms through which GGOH may affect glucose homeostasis and bone health in an obese condition. 

Diabetic rats treated with insulin appear to protect hyperglycemia-induced trabecular bone deterioration. De Oliveira et al. reported that streptozotocin-induced hyperglycemia negatively affected the trabecular bone of LV in rats, as shown by decreased BV/TV, Tb.Th, and Tb.N [39]. In the present study, we observed the direct skeletal effects of individual GGOH and GTP supplementation on bone health. The hypoglycemic effects of individual GGOH and GTP supplementation may also contribute to the osteoprotective effects on bone indirectly. However, the combination of GGOH and GTP supplement did not sustain such osteoprotective effects on trabecular bone of obese mice. It is challenging to generalize the results of this single animal study into practice. A future clinical study is warranted to extend the findings from animals to obese individuals on how GGOH and GTP supplementation affects glucose homeostasis and trabecular bone in humans. 

Metformin is commonly used to treat T2DM. Bornstein et al. reported that metformin mitigates the HFD-induced detrimental effects on trabecular and cortical bone of growing mice, via improving osteoblastogenesis and suppressing bone resorption [40]. Although the main focus of this study was to investigate how GGOH and GTP affect glucose homeostasis and bone microstructure, we noted that lack of a positive control, metformin, is a limitation of the study. It is worthy to include metformin as a positive control group in such a factorial design of GGOH and GTP in future mechanistic investigation. 

In conclusion, in an obese male mouse model, individual GGOH and GTP supplementation improved glucose homeostasis, serum CTX, and trabecular microstructure of LV-4 (i.e., Tb.N, Tb.Th, and Tb.Sp). However, the combined GGOH and GTP supplementation compromises such osteoprotective effects on serum CTX and trabecular bone of obese mice. 

## 4. Materials and Methods

### 4.1. Animals and Treatments

Five-week-old male C57BL/6J mice were obtained from Jackson Laboratory, Bar Harbor, ME, USA. After a 5-day acclimation period with chow diet, 48 mice were assigned to 4 groups (n = 12/group) into a 2 (no GGOH vs. 400 mg GGOH/kg diet) × 2 (no GTP vs. 0.5% (*w*/*v*) GTP in drinking water) factorial design, namely a high-fat diet (control). HFD + 400 mg GGOH/kg diet (GG), HFD + 0.5% GTP water (TP), or HFD + GGOH + GTP (GGTP), respectively. In order to improve acceptability for green tea bitterness, 0.1% (weight/volume) Splenda was added into the drinking water for the TP and GGTP groups. Throughout the study, all animals were fed with a high-fat diet (HFD) consisting of 20%, 22% and 58% of energy from carbohydrates, protein, and fat. GGOH with 85% purity was a gift from American River Nutrition, LLC, Hadley, MA, USA. Distilled water mixed with GTP was prepared fresh daily. GTP (decaffeinated with purity 98.5%) (Zhejiang Yixin Pharmaceutical Co., Ltd., Zhejiang, China) consisted of 65.37% epigallocatechin-3-gallate (EGCG), 19.08% epicatechin-3-gallate, 9.87% epicatechin, 4.14% epigallocatechin, and 1.54% catechin. Animals had free access to water and food during the 14-week study period. The mice (3–4 mice/cage) were housed and maintained at a controlled temperature of 21 ± 2 °C with a 12 h light-dark cycle. Our GGOH dosage at 400 mg/kg diet in mice corresponds to approximately 180 mg daily in a 70 kg body weight human. Our GTP dosage at 0.5% weight/volume dosage in mice corresponds to 4–6 cups per day of tea equivalent in human consumption. This study was approved by the Texas Tech University Health Sciences Center Institutional Animal Care and Use Committee (IACUC #15003).

### 4.2. Glucose and Insulin Tolerance Tests 

Twelve weeks after treatments were started, mice were fasted for 4 h and injected intraperitoneally with glucose (2 mg/g body weight) or insulin (1 U/kg body weight; Humulin, Abbott, Chicago, IL, USA) for intraperitoneal glucose tolerance test (ipGTT) or insulin tolerance test (ipITT), respectively. For both ipGTT and ipITT, blood glucose concentrations were measured in the blood drawn from the tail vein prior to (0 min) and at 15, 30, 60, and 120 min after mice were intraperitoneally injected with glucose or insulin. The total area under the curve (AUC) was calculated using the trapezoidal method.

### 4.3. Sample Collection 

At the end of the experiment, animals were fasted for 4 h and blood was collected from mice anesthetized with isoflurane. Pancreases were stored at −80 °C prior to insulin extraction for ELISA or fixed in Z-fix (AnaTech Ltd., Battle Creek, MI, USA) at room temperature and embedded in paraffin for histological assessment. White adipose tissue (gonadal fat) was harvested from epididymal fat pads and weighed for later adipokine analysis. Femur and lumbar vertebrae-4 (LV-4) were harvested and cleaned of adhering soft tissues and stored in 70% ethanol at 4 °C for later analyses. Blood samples were centrifuged at 1500× *g* for 20 min and the serum samples were obtained and kept at −80 °C until analyzed. 

### 4.4. Insulin and Histological Assessment of Pancreatic Tissue

Serum insulin concentrations were quantified using a mouse insulin ELISA kit (EMD Millipore Co., Billerica, MA, USA). Total pancreatic insulin was extracted from the pancreas by acetic acid extraction and insulin content was determined using the same mouse insulin ELISA kit. Pancreatic tissue sections were immunostained, as described previously [41], for insulin and glucagon. Briefly, antigen retrieval was performed by heating slides to a boil (100 °C) for 15 min in 0.01 M sodium citrate buffer (pH 6.0) in a microwave at full power. Slides were then quenched with hydrogen peroxide, blocked with 10% goat serum, and incubated with guinea pig anti-insulin (diluted 1:1000; Dako Agilent Pathology Solutions, Santa Clara, CA, USA) or mouse anti-glucagon (diluted 1:5000; Sigma-Aldrich, St. Louis, MO, USA) primary antibodies. Tissue sections were then incubated with biotinylated secondary antibodies (Vector Laboratories, Burlingame, CA, USA), avidin-biotin-enzyme complex (Vector Laboratories), and diaminobenzidine as chromogen (BioGenex, Fermont, CA, USA). Tissue sections were counterstained with Mayer’s hematoxylin. 

### 4.5. Bone Biomarkers Analyses

Following the manufacturer’s instruction, the concentrations of procollagen type 1 N-terminal propeptide (P1NP) and C-terminal telopeptide of type I collagen (CTX-1) in serum were quantified using respective kits (Immunodiagnostic System Ltd., Scottsdale, AZ, USA). 

### 4.6. Bone Microarchitecture Measurement with μ-CT

The LV-4 and right femur were scanned using μCT (Scanco μCT 40; SCANCO Medical AG, Switzerland) following Cao et al. [42] and the recommended guidelines for μCT scanning [43]. The entire trabecular bone between the cranial and the caudal area of the LV-4 was scanned and analyzed. For femoral trabecular bone, the volume of interest (VOI) comprised 100 cross-sectional slices of the secondary spongiosa starting from about 0.1 mm distal to growth plate. For assessment of cortical indexes, the VOI included 100 slices at the femoral mid-diaphysis between the top of the femur head and the bottom of the lateral and medial condyles. All scans were performed in 1024 × 1024 matrix resulting in an isotropic voxel resolution of 12 μm^3^. An integration time of 300 milliseconds per projection was used. Bone nomenclature was based on Parfitt et al. [44]. Trabecular parameters in both LV-4 and femur included trabecular bone volume per unit bone area/total volume (BV/TV, %), number (Tb.N, 1/mm), separation (Tb.Sp, mm), and thickness (Tb.Th, mm), structure model index (SMI) and trabecular connectivity density (Conn.Dn, mm^−^^3^). Cortical parameters in femur included bone area/total area (B.Ar/T.Ar, mm^2^), medullary area (Me.Ar, mm^2^), and cortical thickness (Ct.Th, mm). The operator performing the scans and analysis was blinded to treatments. 

### 4.7. Adipokine Measurements

White adipose tissue was homogenized with modified radioimmunoprecipitation assay buffer containing protease inhibitors (Sigma-Aldrich, St. Louis, MO, USA). The concentrations of resistin, leptin, monocyte chemoattractant protein-1 (MCP-1), and interleukin (IL)-6 were determined in the adipose tissue homogenate using a multiplexing system (Luminex-MagPix, Luminex Corporation, Austin, TX, USA) and values were normalized to total protein content.

### 4.8. Statistical Analysis 

Results of are presented as mean ± standard error of the mean (SEM) and analyzed by two-way analysis of variance (ANOVA) tests (SigmaStat software v14.0, Systat Software, Inc., San Jose, CA, USA), followed by post hoc Fisher’s Least Significant Difference (LSD) test. The overall effects of GG and GTP on blood ipGTT and ipITT were analyzed by repeated measures ANOVA. Mixed model ANOVA with repeated measures was used to test the differences at each time point for blood ipGTT and ipITT. An alpha of *p*-value < 0.05 applies to all statistical tests. 

## Figures and Tables

**Table 1:** Effect of geranylgeraniol (GGOH) and green tea polyphenols (GTP) on blood glucose and AUC during ipGTT and ipITT, serum insulin concentration, and pancreas insulin concentration.

Parameters	-GGOH		+GGOH		Two-Way ANOVA *p*-Value		
	-GTP (Control)	+GTP (TP)	-GTP (GG)	+GTP (GGTP)	GGOH	GTP	GGOH×GTP
*Blood sugar (mg/dL) measured by ipGTT*							
0 min	186.3 ± 9.5	168.1 ± 9.5	166.6 ± 8.8	169.8 ± 9.5	0.343	0.433	0.263
15 min	506.2 ± 24.6	492.3 ± 24.6	476.0 ± 22.8	483.0 ± 24.6	0.423	0.889	0.671
30 min	542.0 ± 17.8	510.6 ± 17.8	553.1 ± 16.5	540.0 ± 17.8	0.260	0.218	0.609
60 min	512.3 ^a^ ± 34.3	504.7 ^a^ ± 34.3	427.4 ^b^ ± 31.7	439.8 ^b^ ± 34.3	**0.037**	0.945	0.769
120 min	369.3 ^ax^ ± 20.2	255.0 ^ay^ ± 20.2	243.6 ^bx^ ± 18.7	207.3 ^by^ ± 20.2	**<0.001**	**0.001**	0.062
AUC	57125 ^ax^ ± 2009	49374 ^ay^ ± 1835	50496 ^bx^ ± 1835	46680 ^by^ ± 1834	**0.023**	**0.006**	0.308
*Blood glucose (mg/dL)* *measured by ipITT*							
0 min	171.1 ± 7.4	162.7 ± 7.4	161.1 ± 6.9	166.1 ± 6.9	0.651	0.809	0.354
15 min	88.6 ± 7.9	93.1 ± 7.9	95.7 ± 7.3	85.7 ± 7.3	0.979	0.722	0.352
30 min	81.8 ± 5.5	75.7 ± 5.5	81.0 ± 5.1	80.3 ± 5.1	0.724	0.523	0.612
60 min	68.7^x^ ± 6.3	53.0 ^y^ ± 6.3	63.9 ^x^ ± 5.8	53.3 ^y^ ± 5.8	0.713	**0.042**	0.679
120 min	90.8 ^ax^ ± 6.5	74.0 ^ay^ ± 7.1	74.1 ^bx^ ± 6.5	58.9 ^by^ ± 6.0	**0.025**	**0.024**	0.909
AUC	9313 ± 464	8214 ± 430	8143 ± 464	7630 ± 430	0.063	0.085	0.519
Serum insulin (ng/mL)	13.2 ^y^ ± 1.8	37.0 ^x^ ± 1.9	14.0^y^ ± 1.9	36.7^x^ ± 1.8	0.897	**<0.001**	0.762
Pancreas insulin (μg/mL)	341.4 ± 25.4	305.6 ± 27.1	341.0 ± 25.4	334.1 ± 25.4	0.591	0.415	0.579

Different letters (^a^ and ^b^ for GGOH effect; ^x^ and ^y^ for GTP effect) are significantly different by two-way ANOVA (*p* < 0.05). Values are mean (n = 12/group) with the standard error of mean (SEM). Group assignment includes control group (high-fat diet group); TP group (high-fat diet and green tea polyphenols at 0.5% (*v*/*w* in drinking water)); GG group (high-fat diet and geranylgeraniol at 400 mg/kg diet); GGTP (high-fat diet, geranylgeraniol, and green tea polyphenols). AUC, area under the curve.

**Table 2:** Effects of GGOH and GTP supplementation on bone parameters.

Parameters	-GGOH		+GGOH		Two-Way ANOVA *p*-Value		
	-GTP (Control)	+GTP (TP)	-GTP (GG)	+GTP (GGTP)	GGOH	GTP	GGOH×GTP
*Serum bone biomarkers*							
P1NP (ng/mL)	22.3 ± 0.9	20.5 ± 1.0	21.2 ± 0.9	20.6 ± 0.9	0.604	0.227	0.561
CTX (ng/mL)	21.3 ^A^ ± 1.6	14.0 ^B^ ± 1.7	14.2 ^B^ ± 1.6	13.8 ^B^ ± 1.5	**0.031**	**0.022**	**0.041**
*LV-4 (trabecular bone)*							
BV/TV (%)	17.5 ± 0.9	18.1 ± 0.1	18.4 ± 0.1	16.7 ± 0.1	0.814	0.552	0.213
Tb.N (mm^−1^)	5.36 ^b^ ± 0.09	5.40 ^b^ ± 0.09	5.53 ^a^ ± 0.09	5.63 ^a^ ± 0.09	**0.033**	0.450	0.740
Tb.Th (mm)	0.044 ^y^ ± 0.001	0.044 ^x^ ± 0.001	0.041 ^y^ ± 0.001	0.046 ^x^ ± 0.001	0.280	**0.038**	0.110
Tb.Sp (mm)	0.185 ^a^ ± 0.003	0.185 ^a^ ± 0.003	0.176 ^b^ ± 0.003	0.177 ^b^ ± 0.003	**0.019**	0.990	0.948
Conn.Dn (mm^−3^)	167.7 ^C^ ± 10.4	179.4 ^B^ ± 10.9	221.1 ^A^ ± 10.9	186.8 ^B^ ± 10.4	**0.006**	0.295	**0.036**
SMI	1.702 ^A^ ± 0.115	1.622 ^A^ ± 0.120	1.242 ^B^ ± 0.120	1.639 ^A^ ± 0.115	0.067	0.186	**0.049**
*Distal femur (trabecular bone)*							
BV/TV (%)	7.043 ± 0.924	7.255 ± 0.924	9.462 ± 0.850	7.255 ± 0.924	0.189	0.278	0.189
Tb.N (mm^−1^)	3.397 ± 0.146	3.543 ± 0.146	3.663 ± 0.134	3.543 ± 0.146	0.357	0.928	0.357
Tb.Th (mm)	0.048 ± 0.002	0.047 ± 0.002	0.051 ± 0.002	0.047 ± 0.002	0.422	0.199	0.422
Tb.Sp (mm)	0.300 ± 0.014	0.288 ± 0.014	0.270 ± 0.013	0.288 ± 0.013	0.268	0.821	0.268
Conn.Dn (mm^−3^)	28.23 ± 8.26	36.60 ± 8.26	50.43 ± 7.59	36.60 ± 8.26	0.178	0.737	0.178
SMI	2.983 ^A^ ± 0.113	2.911 ^A^ ± 0.113	2.588 ^B^ ± 0.104	2.911 ^A^ ± 0.113	0.080	0.260	0.080
*Femur mid-diaphysis (cortical bone)*							
B.Ar (mm^2^)	0.990 ± 0.031	0.985 ± 0.029	0.981 ± 0.028	1.012 ± 0.029	0.753	0.660	0.556
Me.Ar (%)	51.71 ± 0.64	51.69 ± 0.62	51.83 ± 0.59	50.50 ± 0.61	0.385	0.280	0.297
Ct.Th (mm)	0.212 ± 0.004	0.210 ± 0.004	0.212 ± 0.004	0.216 ± 0.004	0.537	0.813	0.457

Different letters (^a^ and ^b^ for GGOH effect; ^x^ and ^y^ for GTP effect; ^A^ and ^B^ for interaction effect) are significantly different by two-way ANOVA and Fisher’s LSD test (*p* < 0.05). Values are mean (n = 12/group) with the standard error of mean (S.E.M.). Group assignment includes control group (high-fat diet group); TP group (high-fat diet and green tea polyphenols at 0.5% (*v*/*w* in drinking water)); GG group (high-fat diet and geranylgeraniol at 400 mg/kg diet); GGTP (high-fat diet, geranylgeraniol, and green tea polyphenols). Abbreviation: geranylgeraniol, GGOH; green tea polyphenols, GTP; BV//TV, bone volume/total volume, B.Ar, cross-sectional bone area; Conn.Dn, connectivity density; Ct.Th; cortical thickness, Me.Ar, cross-sectional marrow area; T.Ar, cross-sectional total area; Tb.N, trabecular number; Tb.Sp, trabecular separation; Tb.Th, trabecular thickness; SMI, structure model index.

**Table 3:** Effects of GGOH and GTP supplementation on final body weights, white adipose tissue weights and adipokine concentrations.

Parameters	-GGOH		+GGOH		Two-Way ANOVA *p*-Value		
	-GTP (Control)	+GTP (TP)	-GTP (GG)	+GTP (GGTP)	GGOH	GTP	GGOH×GTP
Final BW	37.9 ^ax^ ± 1.30	34.3 ^ay^ ± 1.40	35.3 ^bx^ ± 1.40	31.5 ^by^ ± 1.40	**0.049**	**0.009**	0.960
WAT weight (g)	2.23 ^ax^ ± 0.16	1.75 ^ay^ ± 0.16	1.90 ^bx^ ± 0.16	1.36 ^by^ ± 0.16	**0.031**	**0.003**	0.870
Resistin (pg/mg protein)	253.4 ^x^ ± 43.2	63.5 ^y^ ± 4.4	238.4 ^x^ ± 26.5	149.0 ^y^ ± 17.8	0.202	**<0.001**	0.073
Leptin (pg/mg protein)	37.8 ^x^ ± 8.1	5.7 ^y^ ± 1.4	29.8 ^x^ ± 3.4	10.4 ^y^ ± 1.8	0.712	**<0.001**	0.176
IL-6 (pg/mg protein)	0.662 ^x^ ± 0.106	0.194 ^y^ ± 0.021	0.839 ^x^ ± 0.176	0.321 ^y^ ± 0.040	0.141	**<0.001**	0.701
MCP-1 (pg/mg protein)	0.413 ^x^ ± 0.067	0.023 ^y^ ± 0.007	0.327 ^x^ ± 0.066	0.140 ^y^ ± 0.077	0.836	**<0.001**	0.174

Different letters (^a^ and ^b^ for GGOH effect; ^x^ and ^y^ for GTP effect) are significantly different by two-way ANOVA (*p* < 0.05). Values are mean (n = 12/group) with the standard error of mean (S.E.M.). Group assignment includes control group (high-fat diet group); TP group (high-fat diet and green tea polyphenols at 0.5% (*v*/*w* in drinking water)); GG group (high-fat diet and geranylgeraniol at 400 mg/kg diet); GGTP (high-fat diet, geranylgeraniol, and green tea polyphenols). Abbreviation: BW, body weight; GGOH, geranylgeraniol; GTP, green tea polyphenols; IL-6, ineterleukin-6; MCP-1, monocyte chemoattractant protein-1; WAT, white adipose tissue.
